# Supplementary figures and images for: Effect of Astaxanthin on Tissue Transglutaminase and Cytoskeletal Protein Expression in Amyloid-Beta Stressed Olfactory Ensheathing Cells: Molecular and Delayed Luminescence Studies
Source: Antioxidants (Basel). 2023 Mar 19;12(3):750. doi: 10.3390/antiox12030750 (PMC10045022; doi:10.3390/antiox12030750)

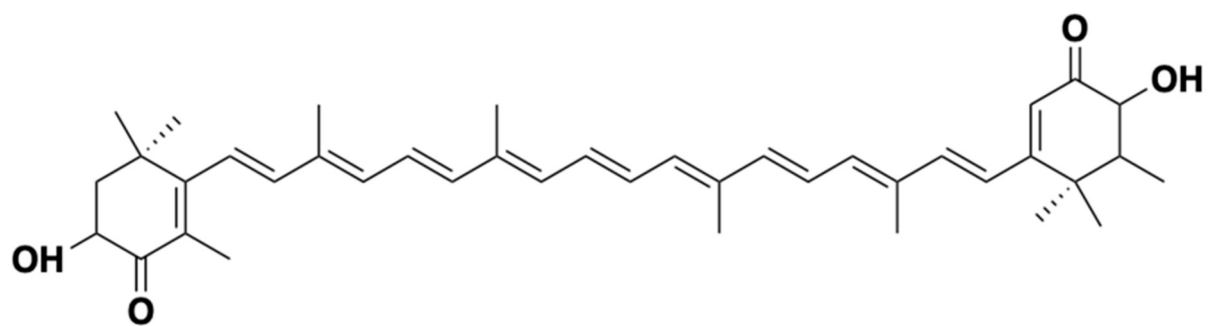

**Figure S1.** Astaxanthin structure.

Supplement: Supplementary file 1 [file antioxidants-12-00750-s001.zip › antioxidants-2289195-supplementary.pdf]
